# PANoptosis components, regulation, and implications

**DOI:** 10.18632/aging.103528

**Published:** 2020-06-19

**Authors:** R.K. Subbarao Malireddi, Rebecca E. Tweedell, Thirumala-Devi Kanneganti

**Affiliations:** 1 Department of Immunology, St. Jude Children's Research Hospital, Memphis, TN 38105, USA

**Keywords:** PANoptosis, ZBP1, pyroptosis, apoptosis, necroptosis, inflammatory cell death

The innate immune system senses cellular stressors and microbial infections to activate programmed cell death (PCD) pathways. Pyroptosis, apoptosis, and necroptosis are three key PCD pathways characterized by their molecular and genetic features. Dysregulation of these pathways promotes disease, including aging-related autoimmune and neurodegenerative diseases and cancer. While early studies of cell death focused on the unique genetic programs and biochemical functions that comprise each of these individual mechanisms, recent studies indicate remarkable crosstalk and redundancies among them. Our studies have connected the inflammasome sensors, caspase-1, and caspase-11 (components of pyroptosis) with caspase-8, caspase-7, and PARP (components of apoptosis), and RIPK1 and RIPK3 (components of necroptosis). These discoveries laid the foundation for us to pioneer the concept of PANoptosis [1], which we define as the integration of the pyroptosis, apoptosis, and necroptosis pathways into a unified mechanism of inflammatory cell death. Understanding the regulation and evolutionary relevance of PANoptosis in health and disease is key to identifying ways to globally modulate these processes for disease prevention and treatment.

We have sought to identify master regulators that act as central hubs to control assembly of ripoptosome-like multifaceted cell death complexes (PANoptosomes) which drive PANoptosis [1–5]. Transforming growth factor beta-activated kinase 1 (TAK1), typically involved in prosurvival signaling, causes cells to undergo PANoptosis when it is inactivated or deleted [2,3]. Z-DNA binding protein 1 (ZBP1), a unique innate sensor, is essential for detecting influenza A virus and activating the NLRP3 inflammasome and PANoptosis [4]. Several other molecules can also regulate PANoptosis, including proline-serine-threonine phosphatase-interacting protein 2 (PSTPIP2), A20, sharpin, and others. Characterization of these molecules indicates the existence of unique master regulators and fundamentally different mechanisms to drive PANoptosis. However, studies focused on comprehensively elucidating the role and regulation of PANoptosis are lagging behind.

Our studies of TAK1 have characterized the roles of several crucial PANoptotic molecules [2,3]. In the absence of external stimuli, TAK1 deficiency causes loss of cellular homeostasis and unleashes RIPK1 kinase activity-dependent inflammatory signaling, NLRP3 inflammasome activation, and PANoptosis. This multifaceted inflammatory cell death is triggered by the formation of a PANoptosome containing RIPK1, ASC, and caspase-8 that promotes FADD-caspase-8–dependent apoptosis, necroptosis through RIPK3-mediated phosphorylation of MLKL, and NLRP3 inflammasome activation and pyroptosis [2]. TAK1-deficient mice have neutrophilia, exhibiting an AML-like phenotype, and are hypersusceptible to inflammatory septic shock; inactivation of RIPK1 kinase activity to inhibit PANoptosis partially protects these mice [2,3].

In addition to our original findings demonstrating RIPK1 kinase activity-dependent PANoptosis, we recently discovered an alternative pathway. In the evolutionary arms race between pathogens and hosts, pathogens such as *Yersinia* inhibit TAK1 in an attempt to evade the host immune response, but hosts have evolved to sense these inhibitory mechanisms and a diverse array of pathogen-associated molecular patterns to trigger multiple modes of cell death and inflammation [1,2,6,7]. We found that when TAK1 is inactivated, Toll-like receptor-mediated innate immune priming relieves the requirement for RIPK1 kinase activity to drive NLRP3 inflammasome activation and PANoptosis [2]. This discovery was unexpected and disproves the dogma that RIPK1 kinase activity is required for cell death. Our findings suggest that macrophages have evolved parallel mechanisms to induce complementary modes of cell death. It is also possible that when TAK1 is present, it actively blocks cell death, enabling immune cells to survive long enough to upregulate anti-microbial molecules that help maintain effective immune function in the local environment.

Mutations in the components of PANoptosis are associated with several diseases, including infectious, inflammatory, neurodegenerative, and metabolic diseases and cancer. Therefore, the paradigm of PANoptosis has far-reaching implications. In infectious disease, activating alternate modes of inflammatory cell death may be more effective at preventing pathogen evasion of immune responses. PANoptosis seems to occur on a pathogen-specific basis, which may be predicated on the ability of individual pathogens that are highly virulent to evade the frontline innate immune defense. As a therapeutic approach, reprogramming the natural cell death mechanism to PANoptosis may help effectively activate immune responses against these pathogens. However, this must be balanced, as excessive PANoptosis can lead to pathological responses and contribute to the development of inflammatory diseases.

In the context of cancer, triggering PANoptosis may have extensive applications to kill a diverse array of cancer cells while simultaneously activating lasting immune protection. Uncontrolled cellular division is central to cancer progression and can be a consequence of a failure of cancer cells to undergo cell death or immune suppression. While several therapies targeting apoptosis have shown some clinical success, cancer cells frequently develop resistance through mutations that bypass the apoptotic pathway. Promoting PANoptosis has the potential to overcome this aberration and initiate robust inflammatory cell death that primes the immune system while decreasing the chances of developing resistance. Additionally, acute activation of PANoptosis could complement existing cancer immunotherapies. Historically, successful approaches have been largely based on cytotoxic T-cell function, but many patients’ responses are hindered by dysfunctional T cells. Harnessing the potent inflammatory immune response of PANoptosis may potentiate T-cell functionality and durability in the tumor microenvironment, improving therapeutic outcomes.

Furthermore, PANoptosis can contribute to autoinflammation, neuroinflammation, and metabolic inflammation, causing wide-spread effects throughout the body. The release of proinflammatory cytokines and other damage-associated molecular patterns driven by PANoptosis likely contributes to this phenomenon. Common mechanistic themes underlying several age-associated diseases include loss of cellular homeostasis and low-grade inflammatory responses, and TAK1 deficiency seems to substantially contribute to these events. Indeed, a recent study found that reduced expression of TAK1 due to aging causes RIPK1-driven neurodegeneration [8]. These findings clearly demonstrate that modulation of key regulators of PANoptosis may help prevent inflammatory diseases.

Overall, the process of PANoptosis is implicated in many diverse diseases and deserves further study to inform the development of new and improved therapeutic strategies.

## References

[1] Malireddi RK, et al. Front Cell Infect Microbiol. 2019; 9:406. 10.3389/fcimb.2019.00406 31850239 PMC6902032

[2] Malireddi RK, et al. J Exp Med. 2020; 217:e20191644. 10.1084/jem.20191644 31869420 PMC7062518

[3] Malireddi RK, et al. J Exp Med. 2018; 215:1023–34. 10.1084/jem.20171922 29500178 PMC5881469

[4] Kuriakose T, et al. Sci Immunol. 2016; 1:aag2045. 10.1126/sciimmunol.aag2045 27917412 PMC5131924

[5] Lukens JR, et al. Nature. 2014; 516:246–49. 10.1038/nature13788 25274309 PMC4268032

[6] Orning P, et al. Science. 2018; 362:1064–69. 10.1126/science.aau2818 30361383 PMC6522129

[7] Sarhan J, et al. Proc Natl Acad Sci USA. 2018; 115:E10888–97. 10.1073/pnas.1809548115 30381458 PMC6243247

[8] Xu D, et al. Cell. 2018; 174:1477–1491.e19. 10.1016/j.cell.2018.07.041 30146158 PMC6128749

